# Association between *KCNJ6* (*GIRK2*) Gene Polymorphisms and Postoperative Analgesic Requirements after Major Abdominal Surgery

**DOI:** 10.1371/journal.pone.0007060

**Published:** 2009-09-16

**Authors:** Daisuke Nishizawa, Makoto Nagashima, Ryoji Katoh, Yasuo Satoh, Megumi Tagami, Shinya Kasai, Yasukazu Ogai, Wenhua Han, Junko Hasegawa, Naohito Shimoyama, Ichiro Sora, Masakazu Hayashida, Kazutaka Ikeda

**Affiliations:** 1 Division of Psychobiology, Tokyo Institute of Psychiatry, Tokyo, Japan; 2 Department of Surgery, Toho University Sakura Medical Center, Sakura, Japan; 3 Department of Anesthesiology, Toho University Sakura Medical Center, Sakura, Japan; 4 Department of Anesthesiology and Palliative Medicine, National Cancer Center, Tokyo, Japan; 5 Department of Biological Psychiatry, Tohoku University Graduate School of Medicine, Sendai, Japan; 6 Department of Anesthesiology, Saitama Medical University International Medical Center, Hidaka, Japan; Dr. Margarete Fischer-Bosch Institute of Clinical Pharmacology, Germany

## Abstract

Opioids are commonly used as effective analgesics for the treatment of acute and chronic pain. However, considerable individual differences have been widely observed in sensitivity to opioid analgesics. We focused on a G-protein-activated inwardly rectifying potassium (GIRK) channel subunit, GIRK2, that is an important molecule in opioid transmission. In our initial polymorphism search, a total of nine single-nucleotide polymorphisms (SNPs) were identified in the whole exon, 5′-flanking, and exon-intron boundary regions of the *KCNJ6* gene encoding GIRK2. Among them, G-1250A and A1032G were selected as representative SNPs for further association studies. In an association study of 129 subjects who underwent major open abdominal surgery, the A/A genotype in the A1032G SNP and -1250G/1032A haplotype were significantly associated with increased postoperative analgesic requirements compared with other genotypes and haplotypes. The total dose (mean±SEM) of rescue analgesics converted to equivalent oral morphine doses was 20.45±9.27 mg, 10.84±2.24 mg, and 13.07±2.39 mg for the A/A, A/G, and G/G genotypes in the A1032G SNP, respectively. Additionally, *KCNJ6* gene expression levels in the 1032A/A subjects were significantly decreased compared with the 1032A/G and 1032G/G subjects in a real-time quantitative PCR analysis using human brain tissues, suggesting that the 1032A/A subjects required more analgesics because of lower *KCNJ6* gene expression levels and consequently insufficient analgesic effects. The results indicate that the A1032G SNP and G-1250A/A1032G haplotype could serve as markers that predict increased analgesic requirements. Our findings will provide valuable information for achieving satisfactory pain control and open new avenues for personalized pain treatment.

## Introduction

Opioids are commonly used as effective analgesics for the treatment of acute and chronic pain. However, sensitivity to opioid analgesics is well known to vary widely among individual subjects [1]. Individual differences can be attributed to both genetic and environmental factors, although the relative influence of each of these factors can be diverse [2]. Genetic variations in opioid-related genes involved in opioid pharmacokinetics and pharmacodynamics might lead to individual differences in phenotypes related to pharmacological actions of opioid analgesics.

Numerous molecules are involved in the pharmacological effects of opioids. Opioid ligands bind to opioid receptors, and the signal is transmitted to a variety of effectors (e.g., adenylate cyclase, calcium ion channels, and G-protein-activated inwardly rectifying potassium [GIRK] channels), thereby resulting in analgesic effects [3]. The genes encoding these molecules are candidates for researching the relationships between genetic variations and individual differences in phenotypes related to opioid actions.

Among opioid-related genes, GIRK channels are attractive targets for the investigation of the relationship between genetic variations and sensitivity to opioid analgesics because they play a key role in opioid-induced analgesia [3]. Additionally, recent quantitative trait locus analysis and computational mapping have identified *Kcnj9* (mouse *Girk3*) as a candidate gene affecting variability in the analgesia induced by multiple drug classes [4]. GIRK channels are members of the inwardly rectifying potassium channel family, and four subtypes (GIRK1-GIRK4) have been identified in mammals [5]. GIRK channels are expressed in many tissues, including the heart [6], spinal cord [7], [8], and various regions in the brain with different subunit compositions [9]–[11]. GIRK channel activation is triggered by activation of several G_i/o_ protein-coupled receptors, including opioid receptors [12]. Several studies using knockout mice have shown that opioid-induced GIRK channel activation co-expressed with opioid receptors leads to inhibition of nociceptive transmission and thus opioid-induced analgesia [6], [7], [13]–[15].

To date, however, few studies have examined the relationship between genetic variations in GIRK channels and phenotypic differences in humans, although several studies have identified human GIRK channel gene polymorphisms [16]–[18]. Therefore, the present study focused on GIRK channel gene polymorphisms, particularly those of the *KCNJ6* gene encoding GIRK2 because it has been investigated more extensively than the other subtypes with regard to its involvement in analgesia [6], [7], [13]–[15]. We sought to reveal the relationship between genetic variations in the *KCNJ6* gene and individual differences in opioid analgesic sensitivity.

## Methods

### Ethics Statement

The study protocol was approved by the Institutional Review Boards at the Institute of Medical Science, The University of Tokyo (Tokyo, Japan), Toho University Sakura Medical Center (Sakura, Japan), and the Tokyo Institute of Psychiatry (Tokyo, Japan). All subjects provided informed, written consent for the genetics studies.

### Subjects

Subjects for the resequencing of the *KCNJ6* gene were recruited from the Kanto area in Japan. A total of 48 unrelated healthy subjects were used in the study so that polymorphisms with allele frequency more than approximately 1% could be detected. The oral mucosa of the participants was collected for gene analysis.

The subjects used in the association study were 129 patients who underwent major open abdominal surgery, mostly gastrectomy for gastric cancer and colectomy for colorectal cancer, under combined general and epidural anesthesia at Research Hospital, Institute of Medical Science, The University of Tokyo, or at Toho University Sakura Medical Center. Peripheral blood or oral mucosa samples were collected from these subjects for gene analysis.

To examine *KCNJ6* gene expression levels, a total of 105 human DNA samples extracted from human occipital cortex and 100 RNA samples extracted from human anterior cingulate cortex of the same specimens were additionally obtained from the Stanley Medical Research Institute (Bethesda, MD) as samples independent of that in the association study (SMRI samples).

### Clinical data

Postoperative pain was managed primarily with continuous epidural analgesia with fentanyl or morphine. Fentanyl or morphine was diluted with 0.25% bupivacaine in a total volume of 100 ml and infused at a constant rate of 2 ml/h through a catheter placed in the lower thoracic or upper lumbar epidural space. Whenever the patient complained of significant postoperative pain despite continuous epidural analgesic, appropriate doses of opioids, including morphine, buprenorphine, pentazocine, and pethidine, and/or nonsteroidal anti-inflammatory drugs (NSAIDs), including diclofenac and flurbiprofen, were administered as rescue analgesics at the discretion of surgeons. The clinical data sampled included age, gender, body height, body weight, postoperative diagnosis, type of operation, duration of operation, and doses of rescue analgesics (opioids and/or NSAIDs) administered during the first 24 h postoperative period, for which analgesic therapy would be required in most patients. The study subjects were also asked to rate their pain intensity at rest during the first 24 h postoperative period using a 5-point verbal numerical rating scale (NRS; 0 = no pain, 1 = mild pain, 2 = moderate pain, 3 = severe pain, 4 = extremely severe pain).

To allow intersubject comparisons of rescue analgesic doses required during the first 24 h postoperative period, doses of opioids and NSAIDs administered as rescue analgesics during this period were converted to the equivalent dose of oral morphine according to a previous report [19]. The conversion factor used for the different analgesics to derive equivalent doses of oral morphine is presented in Table 1. The frequency of rescue analgesic administration was determined as the frequency of use of rescue analgesics during the first 24 h postoperative period. The total dose of rescue analgesics administered was calculated as the sum of oral morphine-equivalent doses of all opioids and NSAIDs administered to patients as rescue analgesics during the same period.

**Table 1:** Estimated systemic dose equipotent to 90 mg oral morphine (mg).

Analgesics	Dose	Reference
Morphine (oral)	90	[39]
Morphine (intravenous)	30	[39]
Morphine (epidural)	6	[39], [40]
Pentazocine	90	[39], [41]
Buprenophine	1	[39], [41]
Petidine	360	[39], [42]
Fentanyl	0.3	[39], [43]
Diclofenac	300	[39], [43]–[45]
Flurbiprofen	300	[39], [43]–[47]
Indomethacin	300	[39], [43]–[47]

### Resequencing KCNJ6 and SNP selection for the association study

**Figure 1:**
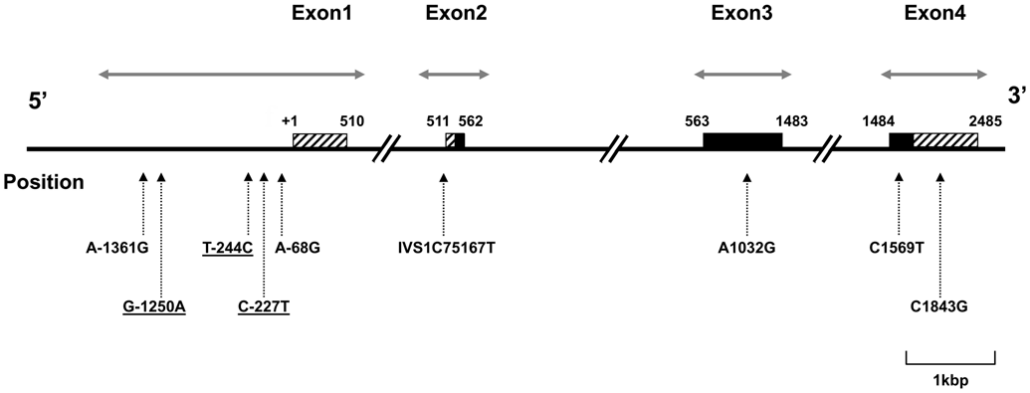
The position of the SNPs identified in the polymorphism screening for the *KCNJ6* gene. The filled box and striped box represent the coding region and untranslated region, respectively. The horizontal arrows indicate the screened regions. The numbers above the boxes and in the exonic SNPs are relative positions from the transcription start site (+1) in the *KCNJ6* mRNA, and the number “75167” in the IVS1C75167T SNP is the relative position from the intron 1 start site in the genomic DNA. The underlined SNPs show absolute linkage disequilibrium between one another (*D'* = 1, *r^2^* = 1).

To comprehensively screen polymorphisms in the *KCNJ6* gene, resequencing was performed using an ABI PRISM® 3100 Genetic Analyzer (Life Technologies Japan Ltd., Tokyo, Japan) for the human *KCNJ6* (*GIRK2*) gene regions (mapped to 21q22.13–q22.2) and 5′-flanking region based on the nucleotide sequences of the GenBank database (accession number: NT_011512). The screened regions contained all consensus sequences of exon regions, exon-intron boundary regions (approximately 30 bp), and putative promoter regions (approximately 1.8 kbp) for the gene. The total length of the screened regions approximately amounted to 4.5 kbp (Figure 1). All primers used for the screening are shown in Table 2.

**Table 2:** Primers used in the analyses.

Gene	Region	Sequence	Forward/Reverse	Primer No.
*KCNJ6*	5′-flanking	TCCCAGTTGCAGTGGACAGGAC	Forward	P1F
		AAATCCCCGGTTAGGAGAAAAGTG	Reverse	P2R
		CCAGTTATTGAAAGGGCCATTATA	Forward	P3F
		CTAAGTAAGTTATTCCCGGAGAAA	Reverse	P4R
		CAGGCATTGTGGAGCACGTATTAC	Forward	P5F
		CACCCCCTCTTTTTCTTATGGTCA	Reverse	P6R
		AATGGGATCCATCTCATTCAACAC	Forward	P7F
		AGAAGGCTTACGGAAACCTCTTAT	Reverse	P8R
	Exon 1	CGGCGGGGTGGGCGTCTC	Forward	P9F
		CCCCCGTGCGAGTTTCAGTCG	Reverse	P10R
		TCGCCCCCGCCCCTTCCT	Forward	P11F
		TCCCTCGCCTTTCGGCTGACTTG	Reverse	P12R
	Exon 2	TTTTGAAAACTGGTCGTGCGTCAC	Forward	P13F
		TTCTGTCTGAAATTTCTGAACG	Reverse	P14R
	Exon 3	AAGTCAACTAGAGGCCTATCCAGA	Forward	P15F
		CTAAGGTCCCCTACCCGGAACATC	Reverse	P16R
		CTGGTGGGCAGGATGGTGAA	Forward	P17F
		TCTCTGCCCTCTTCTTGGGTTGAG	Reverse	P18R
		TTGATCGCATACATACGGGGAGAC	Forward	P19F
		GTCATGAAGCAAGGGGATGTTGTC	Reverse	P20R
		TGGCTACCGGGTCATCACAGATAA	Forward	P21F
		GGCTTCTTGGTGGATATACTTCAG	Reverse	P22R
		TTCTCAATAGAGACAGAAACCACCATTGGT-	Forward	P23F
		TATGGCTACCGGGTCATCACAGATAAATGT		
		GACACCAGAAACAGACGGTCATC	Reverse	P24R
		GGATGAACTCCCCCTCCGAGGTCT	Reverse	P25R
	Exon 4	ACCTACTAAGTGTGGCATCGTATG	Forward	P26F
		AACACATGCAGGTAAGTAACTGAA	Reverse	P27R
		CCCTAGCTGGGCAAACCCTTCTC	Forward	P28F
		TTCCCCCAGACCTATGGCTTGTTG	Reverse	P29R
		TGTGGCAAACCTGGAGAATGAATC	Forward	P30F
		GATCCGTGTGGGAACAGTGAGGTA	Reverse	P31R

Forward/Reverse, sense/antisense strand sequences of the gene, respectively; Primer No., the ID number of the primer described in the paper.

Single-nucleotide polymorphisms (SNPs) for the association studies were selected based on several factors, including recently advanced tagging strategies [20]–[22]. To identify relationships between the SNPs identified in the polymorphism screening, linkage disequilibrium (LD) analysis was performed using Haploview v. 3.32 [23]. For estimation of LD strength between the SNPs, the commonly used *D′* and *r^2^* values were pairwise calculated using the genotype dataset of each SNP. LD blocks were defined among the SNPs showing “strong LD,” based on the default algorithm of Gabriel et al. [24], in which the upper and lower 95% confidence limits on *D′* for strong LD were set at 0.98 and 0.7, respectively. Tag SNPs in the LD block were consequently determined by the software package Tagger, which is incorporated in Haploview and has been detailed in a previous report [22].

### Genotyping

Total genomic DNA was extracted from peripheral blood or oral mucosa samples by standard procedures.

For genotyping *KCNJ6* G-1250A, the polymerase chain reaction-restriction fragment length polymorphism (PCR-RFLP) method and direct sequencing were adopted. To perform PCR-RFLP, the restriction enzyme BsmI (Toyobo Co., Ltd., Tokyo, Japan) and two primers of P5F and P6R were used (Table 2). First, PCR was performed in a final volume of 10 µl containing 5×GoTaq™ reaction buffer (7.5 mM magnesium), 0.16 mM dioxyribonucleoside triphosphate (dNTP), 0.4 µM of each primer, 0.5 U GoTaq™ DNA polymerase (Promega K.K. Japan, Tokyo, Japan), and 5–50 ng extracted genomic DNA as the template. The PCR program was the following: 95°C for 2 min, followed by 35 cycles of 95°C for 30 s, 60°C for 30 s, and 72°C for 1 min, with a final extension at 72°C for 8 min. The amplified DNA fragments were digested by the restriction enzyme at 65°C in a total of 15 µl reaction solution containing 10×M buffer (100 mM Tris-HCl, pH 7.5, 100 mM MgCl_2_, 500 mM NaCl, 10 mM dithiothreitol), 0.3 U BsmI, and 3.5 µl PCR product as the substrate. The digestion products were analyzed by electrophoresis using 1–2% agarose gel and ethidium bromide staining for visualization under ultraviolet illumination. The appearance of only the 601 bp DNA fragment corresponded to the A/A genotype of the loaded sample. The appearance of both the 233 bp and 370 bp fragments corresponded to the G/G genotype, and the appearance of all three 601 bp, 233 bp, and 370 bp DNA fragments corresponded to the G/A genotype. The failure rate of the RFLP genotyping assays was 1.667%.

For genotyping *KCNJ6* A1032G, the PCR-RFLP method, TaqMan allelic discrimination assay (Life Technologies Japan Ltd.) and direct sequencing were adopted. To perform PCR-RFLP, the restriction enzyme BspEI (New England Biolabs, Inc., Ipswich, MA) was used. The forward primer P23F and the reverse primer P24R were used (Table 2). First, PCR was performed in a final volume of 10 µl containing 5×GoTaq™ reaction buffer (7.5 mM magnesium), 0.16 mM dNTP, 0.4 µM of each primer, 0.5 U GoTaq™ DNA polymerase (Promega K.K. Japan, Tokyo, Japan), and 5–50 ng extracted genomic DNA as the template. The PCR program was the following: 95°C for 2 min, followed by 35–40 cycles of 95°C for 30 s, 50°C for 30 s, and 72°C for 1 min, with a final extension at 72°C for 8 min. The amplified DNA fragments were digested by the restriction enzyme at 37°C in a total of 10 µl reaction solution containing 10×NEBuffer 3 (500 mM Tris-HCl, pH 7.9, 100 mM MgCl_2_, 1000 mM NaCl, 10 mM dithiothreitol), 0.5 U BspEI, and 5 µl PCR product as the substrate. The digestion products were analyzed by electrophoresis using 1–2% agarose gel and ethidium bromide staining for visualization under ultraviolet illumination. A 65 bp digested DNA fragment is not easily distinguishable; therefore, the appearance of 395 bp, 332 bp, and both 395 bp and 332 bp DNA fragments corresponded to the A/A, G/G, and A/G genotypes, respectively, of the loaded sample. The failure rate of the RFLP genotyping assays was 3.571%. To perform the TaqMan allelic discrimination assay with a LightCycler 480 (Roche Diagnostics K.K., Tokyo, Japan), TaqMan® SNP Genotyping Assays (Life Technologies Japan Ltd.) containing sequence-specific forward and reverse primers to amplify the polymorphic sequence and two probes labeled with VIC® and FAM™ dye to detect both alleles of the *KCNJ6* A1032G (Assay ID: C_15868122_10) were used. Real-time PCR was performed in a final volume of 10 µl containing 2×LightCycler® 480 Probes Master (Roche Diagnostics K.K.), 40×TaqMan® Gene Expression Assays, 5 ng genomic DNA as the template, and up to 10 µl H_2_O equipped with 2×LightCycler® 480 Probes Master. The thermal condition was the following: 95°C for 10 min, followed by 45 cycles of 95°C for 10 s and 60°C for 60 s, with a final cooling at 50°C for 30 s. Afterward, endpoint fluorescence was measured for each sample well, and the A/A, A/G, and G/G genotypes were determined based on the presence or absence of each type of fluorescence.

For samples that were difficult to genotype for *KCNJ6* G-1250A and *KCNJ6* A1032G using the PCR-RFLP method, direct sequencing was adopted to determine the sequence with both forward and reverse primers enclosing the SNP sites.

### Real-time quantitative PCR (qPCR)

The SMRI RNA samples were treated with DNase I using RNase-Free DNase Set (QIAGEN K.K., Tokyo, Japan) at room temperature (20–25°C) for 10 min, and then clean-up was performed using RNeasy® MinElute® Cleanup Kit (QIAGEN). First-strand cDNA for use in real-time qPCR was synthesized with the SuperScriptIII First-Strand synthesis system for qRT-PCR (Life Technologies Japan Ltd.) with 100 ng purified total RNA according to the manufacturer's protocol.

To perform real-time qPCR utilizing a LightCycler 480 (Roche Diagnostics K.K.), TaqMan® Gene Expression Assays (Life Technologies Japan Ltd.) were used as a probe/primer set specified for the *KCNJ6* gene (Assay ID: Hs01040524_m1) and a probe/primer set for the *ACTB* gene, a house-keeping gene, encoding β-actin (Assay ID: Hs99999903_m1). PCR was performed in a final volume of 20 µl containing 2×LightCycler® 480 Probes Master, 1 µl TaqMan® Gene Expression Assay, 1 µl cDNA as the template, and up to 20 µl H_2_O equipped with 2×LightCycler® 480 Probes Master. The PCR program was the following: 95°C for 10 min, 45 cycles of 95°C for 10 s and 60°C for 30 s, followed by 95°C for 10 s, 50°C for 30 s, 50–70°C (continuously) at a rate 0.06°C/s, with a final cooling at 50°C for 30 s. The expression level of the *KCNJ6* gene was normalized to that of the *ACTB* gene for each sample, and relative *KCNJ6* mRNA expression levels between all samples were compared by setting the lowest expression level among all SMRI samples as 1. Experiments were performed in duplicate (separate experiments) for each sample, and averaged values were calculated for normalized expression levels.

### Predictions of mRNA secondary structure

To discuss in depth mRNA sequence and function, the secondary structure for *KCNJ6* mRNA was predicted using Mfold web server (v. 3.2) with default settings [25]. The *KCNJ6* mRNA position 982–1082 was used to predict the local structure of the mRNA based on the nucleotide sequences of the GenBank database (accession number: NM_002240.2).

### Statistical analysis

The *χ^2^* test or Fisher exact test was performed for all genotype frequency data using FreeJSTAT 8.2 for Windows (free software by M. Sato, Japan; current version is available at http://www.vector.co.jp/soft/win95/business/se030917.html) or Simple Interactive Statistical Analysis (free software by Quantitative Skills, The Netherlands; current version is available at http://www.quantitativeskills.com/sisa/) to investigate the deviation of the distributions from those in the theoretical Hardy-Weinberg equilibrium. Analysis of covariance (ANCOVA) was performed to examine the contribution of the SNPs to the subjective pain ratings reported by patients, frequency of 24 h analgesic requirements, and analgesic requirements during the 24 h postoperative period. Bonferroni multiple comparisons were used as *post hoc* tests. Correction of multiple testing was not performed for the results of the G-1250A and A1032G SNPs in this explorative study. The age, height, and weight of the subjects were incorporated as covariables for the ANCOVA because these three factors were found to be significant covariables in a preliminary analysis and may affect analgesic efficacy of opioids and/or NSAIDs. Pearson's correlation coefficient (*r*) was calculated to examine the correlation between variables. Student's *t*-test was performed to compare *KCNJ6* expression levels between the A1032G genotype subgroups. For these three analyses, SPSS 12.0J for Windows (SPSS Japan, Inc., Tokyo, Japan) was used. Power analyses were performed using G*Power Version 3.0.5 [26]. gPLINK v. 2.049, PLINK v. 1.01 (http://pngu.mgh.harvard.edu/purcell/plink/) [27], and Haploview v. 4.0 [23] were used for haplotype-specific tests, incorporating gender, age, height, and weight of the subjects as covariables, with the false discovery rate set at 0.05 for correction of multiple testing, based on a previous report [28]. In all statistical tests, the criterion for significance was set at *P*<0.05.

## Results

In the first polymorphism search in the whole exon, 5′-flanking, and exon-intron boundary regions of the *KCNJ6* gene, a total of nine SNPs were identified in the 5′-flanking region, intron 1, exon 3, and exon 4. Figure 1 illustrates the relative positions of the SNPs identified in the *KCNJ6* gene. The characteristics of the SNPs are provided in Table 3, where the minor allele frequencies of the SNPs are also shown. The allele frequencies of the SNPs observed in this study were comparable (less than 0.1 difference) to those annotated in the National Center for Biotechnology Information (NCBI) database (Table 3). SNPs for a further association study were selected, considering the LD structure, minor allele frequencies of the SNPs, and the expected impact on gene function. The results of the *D'* and *r^2^* calculations for the *KCNJ6* gene are provided in Table 4. Absolute LD (*D'* = 1, *r^2^* = 1) was observed between SNPs G-1250A, T-244C, and C-227T (Table 4), and Haploview also identified G-1250A as a candidate haplotype-tagging SNP in this LD block structure. The minor allele frequencies for these three SNPs were relatively high (Table 3), and G-1250A was selected for the association study. Among the remaining six SNPs, IVS1C75467T and A1032G are relatively common, with minor allele frequencies greater than 0.1, and could be candidates for an association study. Because IVS1C75467T is in the intron region and thus is less likely to affect the mRNA product or protein levels, it was not selected as a candidate SNP. Therefore, G-1250A and A1032G were selected from the nine *KCNJ6* SNPs for the association study.

**Table 3:** The characteristics of the identified SNPs in the *KCNJ6* gene.

Position	SNP name	rs ID	Sample size	Reported allele	Major allele	Minor allele	Minor allele frequency
5′-flanking	A-1361G	–	48	A	A	G	0.010 (N.A.)
	G-1250A	rs6517442	48	G	A	G	0.385 (0.422)
	T-244C	rs7275707	46	T	C	T	0.391 (0.422)
	C-227T	rs7276069	46	C	T	C	0.391 (0.422)
	A-68G	rs11702683	45	A	G	A	0.089 (0.102)
Intron 1	IVS1C75167T	rs2836016	48	C	T	C	0.188 (0.273)
Exon 3	A1032G	rs2070995	48	A	G	A	0.344 (0.432)
Exon 4	C1569T	rs702859	48	C	T	C	0.062 (0.100)
	C1843G	rs56345212	48	C	C	G	0.052 (N.A.)

rs ID, reference SNP ID in the NCBI dbSNP database; Sample size, the number of samples used for genotyping each SNP; Reported allele, the allele appearing in the GenBank reference sequence. The numbers in parentheses represent the minor allele frequencies for the Japanese population described in the NCBI dbSNP database. N.A., not available.

**Table 4:** Pairwise *D'* and *r^2^* values between the identified SNPs in the *KCNJ6* gene.

*D'*										
	SNP name	A-1361G	G-1250A	T-244C	C-227T	A-68G	IVS1C75167T	A1032G	C1569T	C1843G
	A-1361G	–	1.000	1.000	1.000	1.000	1.000	1.000	1.000	1.000
	G-1250A	0.017	–	***1.000***	***1.000***	1.000	0.136	0.037	0.518	0.159
	T-244C	0.017	***1.000***	–	***1.000***	1.000	0.114	0.020	0.505	0.104
***r^2^***	C-227T	0.017	***1.000***	***1.000***	–	1.000	0.114	0.020	0.505	0.104
	A-68G	0.000	0.153	0.153	0.153	–	0.191	0.398	0.152	0.093
	IVS1C75167T	0.002	0.007	0.005	0.005	0.014	–	0.078	1.000	0.044
	A1032G	0.020	0.000	0.000	0.000	0.034	0.003	–	0.603	0.401
	C1569T	0.001	0.029	0.028	0.028	0.000	0.015	0.046	–	0.091
	C1843G	0.192	0.002	0.001	0.001	0.004	0.000	0.017	0.007	–

The numbers above and below the hyphens show the results of the pairwise calculations of *D'* and *r^2^* between the two SNPs, respectively. The values representing *D'* = 1.000 are underlined. The pairs of values representing *D'* = 1.000 and *r^2^* = 1.000 are italicized and bold.

The genotype distributions for the two SNPs that were selected were not significantly different from the theoretical Hardy-Weinberg equilibrium values in independent tests of the 48 healthy subjects used in the resequencing procedure or 129 patient subjects used in the association analyses (data not shown). The clinical data of the 129 subjects who were included in the association study are provided in Table 5. Rescue analgesics were required in 59 patients. Doses of rescue analgesics administered to patients are shown in Table 5. More detailed clinical data stratified by each genotype (*KCNJ6* G-1250A and A1032G) are presented as Supporting Information in Table S1.

**Table 5:** The clinical data of the subjects included in the study.

	N	Minimum	Maximum	Mean	SD
***KCNJ6*** ** G-1250A**					
G/G (male/female)	19 (11/8)				
G/A (male/female)	72 (42/30)				
A/A (male/female)	35 (19/16)				
***KCNJ6*** ** A1032G**					
A/A (male/female)	11 (5/6)				
A/G (male/female)	62 (37/25)				
G/G (male/female)	56 (32/24)				
**Gender**					
male	74				
female	55				
**Age**	129	28	80	63.57	9.92
**Height (cm)**	129	133	175	158.21	8.34
**Weight (kg)**	129	35	80	56.24	10.42
**NRS pain score**	105	0	4	1.54	1.29
male/female	62/43	0/0	4/4	1.48/1.63	1.21/1.40
**Frequency of analgesic administration**	129	0	6	0.72	1.01
male/female	74/55	0/0	3/6	0.70/0.75	0.90/1.14
**Total dose of rescue analgesics (mg)**	129	0	105	12.63	19.16
male/female	74/55	0/0	72/105	13.38/11.62	19.05/19.44
**Dose of each rescue analgesic (mg)**	129	(0)	(105)	(12.63)	(19.16)
Epidural morphine (mg)	2	0.5 (7.5)	2 (30)	1.25 (18.75)	1.06 (15.91)
Pentazocine (mg)	24	15 (15)	75 (75)	26.25 (26.25)	17.83 (17.83)
Buprenophine (mg)	15	0.2 (18)	0.8 (72)	0.47 (42.00)	0.16 (14.70)
Petidine (mg)	1	35 (8.75)	35 (8.75)	35.00 (8.75)	–
Diclofenac (mg)	14	25 (7.5)	100 (30)	37.50 (11.25)	23.51 (7.054)
Flurbiprofen (mg)	8	50 (15)	100 (30)	62.50 (18.75)	23.15 (6.944)

Values in parentheses for “Dose of each rescue analgesic (mg)” indicate data for dose of each rescue analgesic converted to the equivalent oral morphine dose.

**Figure 2:**
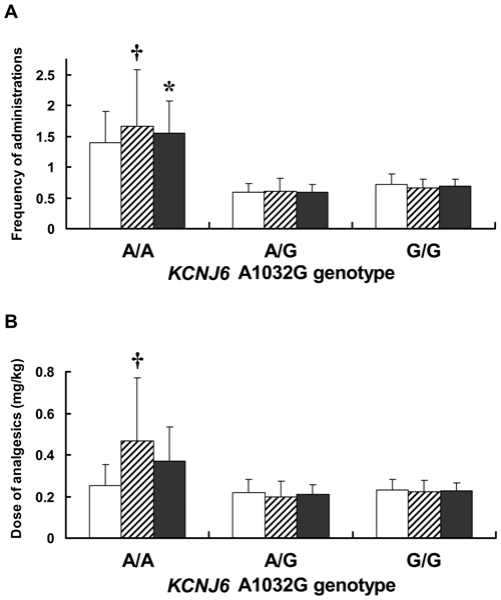
Association analysis between requirements for rescue analgesics and SNPs. The results for the frequency of analgesic administration (A) and the total dose of analgesics administered per weight (B) during the 24 h postoperative period are shown for the *KCNJ6* A1032G SNP. The white, striped, and filled boxes indicate results for male, female, and all subjects, respectively. (A) *: significantly more frequent administration for the A/A genotype compared with the A/G and G/G genotypes in all subjects, †: significantly more frequent administration for the A/A genotype compared with the A/G and G/G genotypes in female subjects. (B) †: significantly greater dose of analgesic administration for the A/A genotype compared with the A/G genotype in female subjects.

Two-way ANCOVA was performed to examine the effects of SNPs and gender on the frequency of rescue analgesic administration, the total dose of rescue analgesics administered, and NRS pain scores, incorporating the age, height, and weight of the subjects as covariables. Statistical power analyses for the ANCOVA revealed that the expected power (1 minus type II error probability) was 71% for the Cohen's conventional “medium” effect size 0.25 [29] when the sample size was 129 and type I error probability was set at 0.05. Significant associations were not observed between *KCNJ6* G-1250A and the frequency of rescue analgesic administration or total dose of rescue analgesics administered (frequency of rescue analgesic administration: *F*_2,117_ = 1.145, *P* = 0.322; total dose of rescue analgesics administered: *F*_2,117_ = 1.233, *P* = 0.295). A significant main effect of *KCNJ6* A1032G on the frequency of rescue analgesic administration was observed (*F*_2,120_ = 5.336, *P* = 0.006). *Post hoc* analysis revealed significant differences between the A/A and A/G genotypes (*P* = 0.005) and between the A/A and G/G genotypes (*P* = 0.010), indicating that the carriers of the A/A genotype in the A1032G SNP required rescue analgesics more often compared with carriers of the A/G and G/G genotypes (Figure 2A). The effect of *KCNJ6* A1032G on the total dose of rescue analgesics administered was not significant (*F*_2,120_ = 1.332, *P* = 0.268). However, a significant main effect of the *KCNJ6* A1032G SNP on the total dose of rescue analgesics administered was observed in the female subjects (*F*_2,49_ = 3.428, *P* = 0.040), and differences were significant between the A/A and A/G genotypes (*P* = 0.040) and marginally significant between the A/A and G/G genotypes (*P* = 0.061) in the *post hoc* analysis (Figure 2B), whereas such differences were not observed in male subjects (*F*_2,68_ = 0.032, *P* = 0.969). Neither the main effect of gender nor the SNP×gender interaction was significant (data not shown). Significant associations were not observed between the two SNPs and NRS pain scores (G-1250A: *F*_2,94_ = 1.455, *P* = 0.239; A1032G: *F*_2,96_ = 0.115, *P* = 0.892), although significant positive correlations were found between NRS pain scores and frequency of rescue analgesic administration (*r* = 0.281, *P* = 0.004) and between NRS pain scores and total dose of rescue analgesics administered (*r* = 0.266, *P* = 0.006), indicating that the patients who received analgesics more frequently felt more pain, possibly attributable to insufficient analgesic effects.

To examine in more detail the association between *KCNJ6* SNPs and rescue analgesic requirements, a haplotype-based test was performed. As shown in Table 6A, a significant association was found between the −1250G/1032A haplotype and the increased frequency of rescue analgesic administration in all patient subjects (*R^2^* = 0.120, adjusted *P* = 0.015). Although no significant associations were observed between each of the *KCNJ6* haplotypes and total dose of rescue analgesics administered in all patient subjects and male subjects (*R^2^* = 0.080, *P* = 0.328; *R^2^* = 0.028, *P* = 0.765, respectively), the −1250G/1032A haplotype was significantly associated with total dose of rescue analgesics administered in female subjects (*R^2^* = 0.277, adjusted *P* = 0.038; Table 6B). Associations between each of the G-1250A/A1032G haplotypes and NRS pain scores were not significant (data not shown).

**Table 6:** Association of *KCNJ6* haplotypes with postoperative analgesia.

**A**						
**Haplotype**	**Frequency**	**Beta**	**R^2^**	**F**	***P***	***P^a^***
−1250G/1032A	0.1517	0.6313	0.1197	8.8080	0.0036^†^	0.0145^*^
−1250A/1032A	0.1736	−0.0216	0.0546	0.0101	0.9203	0.9203
−1250G/1032G	0.2848	−0.0754	0.0563	0.2211	0.6391	0.8521
−1250A/1032G	0.3898	−0.2179	0.0711	2.1290	0.1472	0.2944
**B**						
**Haplotype**	**Frequency**	**Beta**	**R^2^**	**F**	***P***	***P^a^***
−1250G/1032A	0.1394	0.3139	0.2771	7.3160	0.0094^†^	0.0377^*^
−1250A/1032A	0.2032	−0.0160	0.1673	0.0192	0.8903	1.0000
−1250G/1032G	0.2865	−0.0273	0.1687	0.0995	0.7538	1.0000
−1250A/1032G	0.3709	−0.1054	0.1951	1.6770	0.2015	0.8903

Association of the haplotype composed of the G-1250A/A1032G SNPs with (A) the frequency of analgesic administration in all patient subjects or (B) the total dose of analgesics administered during the 24 h postoperative period in female patient subjects. Frequency, haplotype frequency; Beta, regression coefficient; R^2^, coefficient of determination; F, *F* statistic; *P*, crude *P* value; *P^a^*, adjusted *P* value for multiple testing. ^†^ *P*<0.05, ^*^ *P^a^*<0.05.

**Figure 3:**
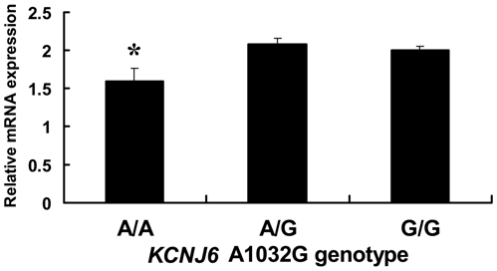
Relative *KCNJ6* mRNA expression level between each genotype subgroup of the SMRI samples. *: significantly lower expression level between the A/A genotype and combined A/G and G/G genotypes in all subjects.

To estimate the impact of the *KCNJ6* A1032G polymorphism on gene expression level, the relative *KCNJ6* mRNA expression level was compared between the genotype subgroups of the SMRI samples in the real-time qPCR. The relative expression level (mean±SEM) was 1.59±0.17, 2.07±0.08, and 1.99±0.06 for the A/A, A/G, and G/G genotypes in the A1032G SNP, respectively (Figure 3), demonstrating that the expression level was 0.76–0.80 fold lower in the A/A genotype than in the A/G and G/G genotype. The difference was significant between the A/A genotype and combined A/G and G/G genotypes (*t*_98_ = 2.265, *P* = 0.026).

## Discussion

The present study comprehensively examined *KCNJ6* genetic variations in humans and explored the associations between these variations and outcomes in clinical pain management. To our knowledge, the present study is the first to explore SNPs of the *KCNJ6* gene with regard to associations between these SNPs and postoperative analgesic requirements in humans. We found that carriers of the A/A genotype in the A1032G SNP or −1250G/1032A haplotype required rescue analgesics more often and tended to require higher doses of rescue analgesics, especially in female subjects, compared with carriers of other genotypes or haplotypes, respectively, after major open abdominal surgery (Figure 2A and B, Table 6). Although we did not show all of the results of multiple testing for the G-1250A and A1032G SNPs with the Bonferroni correction because this study was explorative, the *P* value for the main effect of *KCNJ6* A1032G on the frequency of rescue analgesic administration was 0.012 after the Bonferroni correction. This suggests that this SNP is likely to affect sensitivity to analgesics, although we must concede that this significance might possibly occur by chance alone. Patients who experienced more severe pain, evaluated by NRS pain scores, required higher-dose and more frequent rescue analgesics, although significant associations were not observed between the A1032G SNP or −1250G/1032A haplotype and NRS pain scores. Moreover, *KCNJ6* gene expression levels in the 1032A/A subjects was significantly decreased compared with 1032A/G and 1032G/G subjects in the real-time qPCR analysis using human brain tissues, suggesting that the 1032A/A subjects required more analgesics because of lower *KCNJ6* gene expression levels and consequently insufficient analgesic effects. Altogether, these results suggest that subjects carrying the A/A genotype in the A1032G SNP or −1250G/1032A haplotype, especially in females, had lower sensitivity to analgesics and, therefore, required more rescue analgesics than subjects carrying other genotypes or haplotypes to achieve a similar degree of pain relief.

For our ANCOVA analyses, the desirable sample size was calculated as 158 for the effect size 0.25 to achieve 80% power. This might suggest that a sample size of 129 subjects in our study was somewhat insufficient to reliably detect moderate differences between the SNP genotypes, and a far greater sample may be required to detect smaller differences. Considering this caveat and relatively small effect size observed in the haplotype analysis (e.g., *R^2^* for haplotype effect on the frequency of analgesic administration was 0.120), future studies with larger sample sizes may reveal additional associations between polymorphisms and opioid sensitivity.

In the initial polymorphism screening for *KCNJ6*, a total of nine SNPs were identified in the whole exon, 5′-flanking, and exon-intron boundary regions (Figure 1). Polymorphisms that might cause significant functional changes, such as nonsynonymous or insertion/deletion polymorphisms, were not found in the polymorphism screening of the human *KCNJ6* gene in the present study, possibly attributable to the importance and high conservation of mammalian GIRK channels. A possible explanation derives from studies in *weaver* mice, in which only a single missense mutation in the pore region of the mouse *Kcnj6* gene causes various aberrant changes in cerebellar granule cells [30], membrane permeability [31], loss of K^+^ selectivity [32], [33], significantly lower analgesia compared with wildtype mice [3], and lack of activating effects of ethanol [34]. The *Kcnj6* gene orthologs might be under purifying selection over many generations of the species, including human and mouse, because of the profound functional constraints attributable to the importance of these orthologs in these organisms.

The A/A genotype in the A1032G SNP was significantly associated with increased postoperative analgesic requirements in our study. The G allele appears to be dominant in mediating the transmission of intensified opioid signaling compared with the A allele. However, this particular SNP is synonymous and causes no amino acid change; therefore, the protein structure encoded by this gene may not be altered by this SNP. Nevertheless, local structural difference in the 1023–1059 position was observed between the sequences, including 1032A and 1032G, in our prediction of the *KCNJ6* mRNA secondary structure (Figure S1). Whereas the 1032A mRNA formed an interior loop, a hairpin loop, and a 6 bp helix, the 1032G mRNA formed a bulge loop as well as an interior loop, a hairpin loop, and a 7 bp helix in the local structure. Although the role of this difference in gene function remains to be determined, the SNP may actually influence mRNA expression level. Indeed, recent studies measuring allelic expression imbalances [35] have demonstrated that even a synonymous SNP could affect mRNA and protein levels [36], possibly by altering mRNA stability and protein synthesis [37]. Similar mRNA and protein levels, but altered conformations, were found for synonymous polymorphisms [38].

To further infer the precise mechanism underlying the increased requirements for rescue analgesics in the A/A subjects in the A1032G SNP, we compared the relative *KCNJ6* mRNA expression level between the genotype subgroups of the SMRI samples in the real-time qPCR. A significant difference in expression level was observed between the A/A genotype and the combined A/G and G/G genotypes, consistent with the results of the association study, in which only the subjects with the A/A genotype in this SNP demonstrated significantly higher requirements for rescue analgesics than the other genotypes. The 1032A/A subjects required more analgesics, probably because of lower *KCNJ6* gene expression levels and consequently insufficient analgesic effects.

We do not have any evidence to explain how the −1250G/1032A haplotype contributes to increased requirements for rescue analgesics compared with other haplotypes. One might infer that the G-1250A SNP in the putative regulatory region could be related to some moderate functional alteration, and the −1250G and 1032A alleles could be risk factors for decreased sensitivity to analgesics. Both alleles might combine synergistically to cause profound decreases in sensitivity to analgesics. Future functional studies focusing on both the G-1250A and A1032G SNPs are required to investigate this hypothesis.

In conclusion, the A/A genotype in the *KCNJ6* A1032G SNP and −1250G/1032A haplotype were significantly associated with increased analgesic requirements after major open abdominal surgery. Furthermore, *KCNJ6* gene expression levels in the 1032A/A subjects was significantly decreased compared with the 1032A/G and 1032G/G subjects. Although the association might be restricted to the Japanese population, and the mechanism by which individual sensitivity to postoperative analgesics is altered by the G-1250A and A1032G SNPs remains to be fully elucidated, the outcome indicates that the A1032G SNP and G-1250A/A1032G haplotype could serve as markers that predict increased analgesic requirements. Our findings will provide valuable information to better modulate individual analgesic dosages required to achieve satisfactory pain control and open new avenues for personalized pain treatment.

## Supporting Information

Table S1 The clinical data of the subjects stratified by genotype
(0.12 MB DOC)

Figure S1 Predicted secondary structure for the KCNJ6 mRNA based on the nucleotide sequences of the GenBank database (accession number: NM_002240.2). The sequences for *KCNJ6* mRNA position 1012–1072 are presented for the 1032A (A) and 1032G (B) mRNA. The numbers next to the sequences indicate relative positions from position 982.
(0.99 MB TIF)
