# Supplementary material for: Association between KCNJ6 (GIRK2) Gene Polymorphisms and Postoperative Analgesic Requirements after Major Abdominal Surgery
Source: PLoS One. 2009 Sep 16;4(9):e7060. doi: 10.1371/journal.pone.0007060 (PMC2738941; doi:10.1371/journal.pone.0007060)
Supplement: Table S1 — The clinical data of the subjects stratified by genotype (0.12 MB DOC) [file pone.0007060.s001.doc]

| **Table S1.** |  |  |  |  |  |  |  |
| --- | --- | --- | --- | --- | --- | --- | --- |
| **The clinical data of the subjects stratified by genotype.** | | | |  |  |  |  |
|  |  |  |  |  |  |  |  |
|  |  |  | **N** | **Minimum** | **Maximum** | **Mean** | **SD** |
|  |  |  |  |  |  |  |  |
| **Age** |  |  |  |  |  |  |  |
| *KCNJ6* G-1250A | G/G |  | 19 | 50 | 77 | 61.68 | 7.924 |
|  | G/A |  | 72 | 28 | 80 | 63.00 | 10.886 |
|  | A/A |  | 35 | 47 | 79 | 66.00 | 8.349 |
| *KCNJ6* A1032G | A/A |  | 11 | 52 | 78 | 63.55 | 9.720 |
|  | A/G |  | 62 | 28 | 80 | 64.44 | 9.646 |
|  | G/G |  | 56 | 34 | 80 | 62.61 | 10.334 |
|  |  |  |  |  |  |  |  |
| **Height (cm)** |  |  |  |  |  |  |  |
| *KCNJ6* G-1250A | G/G |  | 19 | 150 | 172 | 159.32 | 6.832 |
|  | G/A |  | 72 | 133 | 172 | 158.55 | 8.755 |
|  | A/A |  | 35 | 141 | 175 | 157.23 | 8.531 |
| *KCNJ6* A1032G | A/A |  | 11 | 136 | 170 | 154.59 | 9.276 |
|  | A/G |  | 62 | 133 | 174 | 157.81 | 8.240 |
|  | G/G |  | 56 | 140 | 175 | 159.38 | 8.168 |
|  |  |  |  |  |  |  |  |
| **Weight (kg)** |  |  |  |  |  |  |  |
| *KCNJ6* G-1250A | G/G |  | 19 | 44 | 77 | 60.11 | 11.709 |
|  | G/A |  | 72 | 35 | 80 | 55.23 | 10.306 |
|  | A/A |  | 35 | 40 | 77 | 56.51 | 10.034 |
| *KCNJ6* A1032G | A/A |  | 11 | 35 | 75 | 57.39 | 11.272 |
|  | A/G |  | 62 | 39 | 77 | 55.31 | 10.302 |
|  | G/G |  | 56 | 38 | 80 | 57.05 | 10.496 |
|  |  |  |  |  |  |  |  |
| **NRS pain score** |  |  |  |  |  |  |  |
| *KCNJ6* G-1250A | G/G |  | 14 | 0 | 3 | 1.43 | 1.222 |
|  | G/A |  | 61 | 0 | 4 | 1.69 | 1.323 |
|  | A/A |  | 28 | 0 | 4 | 1.32 | 1.249 |
| *KCNJ6* A1032G | A/A |  | 5 | 0 | 4 | 2.00 | 1.581 |
|  | A/G |  | 52 | 0 | 4 | 1.58 | 1.319 |
|  | G/G |  | 48 | 0 | 4 | 1.46 | 1.237 |
|  |  |  |  |  |  |  |  |
| **Frequency of analgesic administration** | |  |  |  |  |  |  |
| *KCNJ6* G-1250A | G/G |  | 19 | 0 | 6 | 1.00 | 1.528 |
|  | G/A |  | 72 | 0 | 4 | 0.74 | 0.904 |
|  | A/A |  | 35 | 0 | 3 | 0.51 | 0.853 |
| *KCNJ6* A1032G | A/A |  | 11 | 0 | 6 | 1.55 | 1.753 |
|  | A/G |  | 62 | 0 | 4 | 0.60 | 0.931 |
|  | G/G |  | 56 | 0 | 3 | 0.70 | 0.829 |
|  |  |  |  |  |  |  |  |
| **Total dose of rescue analgesics (mg)** | |  |  |  |  |  |  |
| *KCNJ6* G-1250A | G/G |  | 19 | 0 | 105 | 18.55 | 27.646 |
|  | G/A |  | 72 | 0 | 72 | 12.58 | 17.882 |
|  | A/A |  | 35 | 0 | 60 | 9.72 | 16.534 |
| *KCNJ6* A1032G | A/A |  | 11 | 0 | 105 | 20.45 | 30.757 |
|  | A/G |  | 62 | 0 | 72 | 10.84 | 17.672 |
|  | G/G |  | 56 | 0 | 72 | 13.07 | 17.891 |
|  |  |  |  |  |  |  |  |
