# Supplementary figures and images for: Association between KCNJ6 (GIRK2) Gene Polymorphisms and Postoperative Analgesic Requirements after Major Abdominal Surgery
Source: PLoS One. 2009 Sep 16;4(9):e7060. doi: 10.1371/journal.pone.0007060 (PMC2738941; doi:10.1371/journal.pone.0007060)

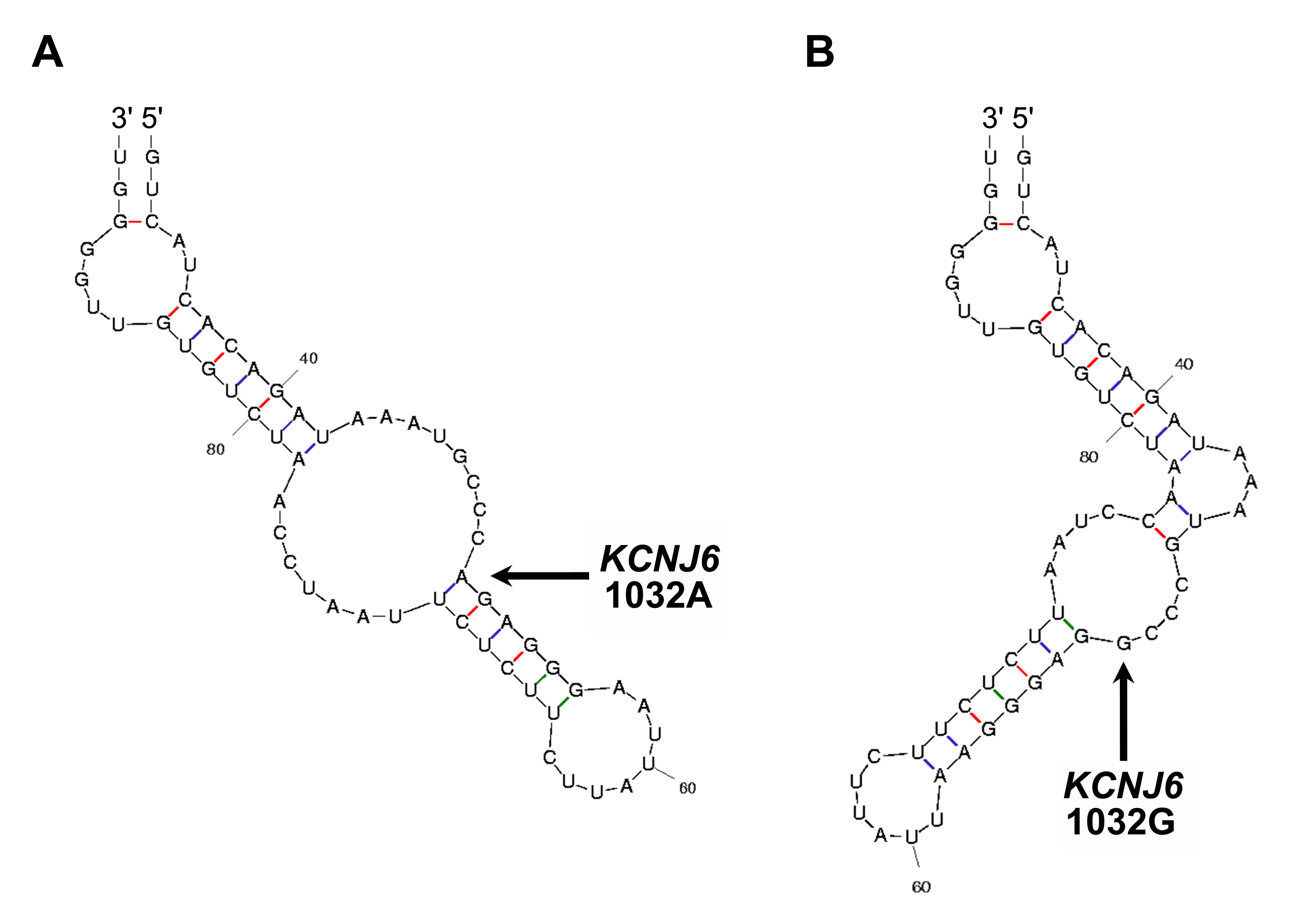

Supplement: Figure S1 — Predicted secondary structure for the KCNJ6 mRNA based on the nucleotide sequences of the GenBank database (accession number: NM_002240.2). The sequences for KCNJ6 mRNA position 1012–1072 are presented for the 1032A (A) and 1032G (B) mRNA. The numbers next to the sequences indicate relative positions from position 982. (0.99 MB TIF) [file pone.0007060.s002.tif]
